# Complete Genome Sequence of Mycobacteriophage Fulbright

**DOI:** 10.1128/MRA.00123-21

**Published:** 2021-03-18

**Authors:** Hari Kotturi, Umar Sahi, Cameron Kedy, Ahmed K. Ali

**Affiliations:** a Department of Biology, University of Central Oklahoma, Edmond, Oklahoma, USA; b Department of Biology, College of Science, Mustansiriyah University, Baghdad, Iraq; DOE Joint Genome Institute

## Abstract

Mycobacteriophage Fulbright was isolated from soil in central Oklahoma using Mycobacterium smegmatis mc^2^115. The genome of phage Fulbright is 42,396 bp long and contains 70 open reading frames (ORFs), with 33 having predicted functions and 37 having hypothetical proteins. It belongs to cluster N and shares 99% nucleotide identity with mycobacteriophage Phloss.

## ANNOUNCEMENT

**FIG 1:**
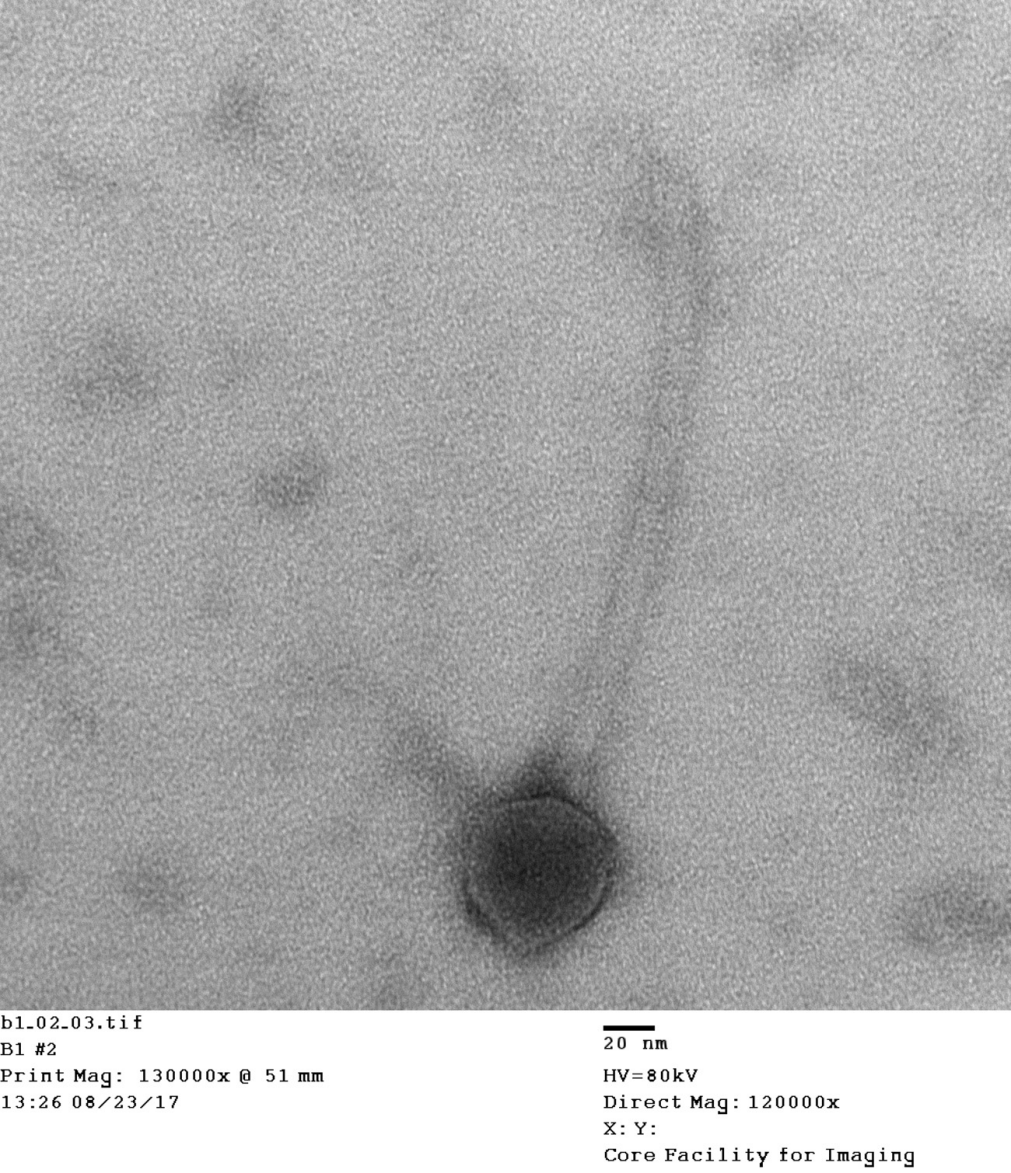
Transmission electron microscopy (TEM) of Fulbright on a Formvar-coated copper grid stained with uranyl acetate, imaged using a Hitachi H-7600 machine.

Mycobacteriophages are bacteriophages that are capable of infecting a mycobacterial host (1). The genus *Mycobacterium* is composed of acid-fast and obligatory aerobic bacteria. Mycobacteria are ubiquitous microorganisms that were isolated from various soil types, such as those found on ranches, landfills, and boreal coniferous forests (2, 3). Mycobacteriophage Fulbright was isolated from a soil sample collected from the campus of the University of Central Oklahoma (UCO) (global positioning system [GPS] coordinates, 35.658889N, 97.474444W). The phage was isolated, purified, and propagated using the host, M. smegmatis mc^2^155, following the protocols described in the Science Education Alliance-Phage Hunters Advancing Genomics and Evolutionary Science (SEA-PHAGES) manual (4). Briefly, the collected soil sample was enriched with M. smegmatis mc^2^155, incubated at 37°C for 24 h, filtered, and plated with the host bacteria. Three plaque purifications were done to purify the phage for imaging and DNA extraction. Transmission electron microscopy images of the phage particle were taken by mounting sample on a carbon-stabilized, Formvar-coated copper grid stained with 1% uranyl acetate solution. Photographs were taken using the Hitachi H-7600 machine. Fulbright has a *Siphoviridae* morphology with an isometric head and a long, flexible noncontractile tail (Fig. 1). Phage genomic DNA was extracted from lysate following the SEA-PHAGES recommended protocols (4) using a Promega Wizard DNA cleanup kit. An Ultra II FS kit with dual-indexed barcoding (New England Biolabs) was used for generating the genomic libraries. The pooled libraries were run on an Illumina MiSeq system to yield single-end 150-base reads. Newbler v.2.9 and Consed v.29 were used with default parameters for assembling the genome and assessing the quality of the assembly (5). The approximate sequencing coverage of the phage genome is 2,501× with 749,000 reads used for the assembly. The assembled phage genome was annotated by students in the bioinformatics course at UCO in the spring of 2019 using the SEA-PHAGES-recommended parameters with DNAMaster v.5.0.2 (http://cobamide2.bio.pitt.edu/computer.htm), GeneMark v.3.25 (6), NCBI BLAST v.2.9.0 (7), Glimmer v.3.02 (8), HHpred v.3.2.0 (9), ARAGORN v.1.2.38 (10), and Phamerator (11).

The genome of phage Fulbright is 42,396 bp long with a 63% G+C content. It has a 3′ sticky end with a 13-bp overhang which was determined as previously described (5). Genome analysis indicated that Fulbright has 70 open reading frames (ORFs), with 33 having predicted functions and 37 having hypothetical proteins. All predicted ORFs are transcribed in the forward direction, except ORFs 24, 25, 32 to 36, and 67. The predicted structural and assembly ORFs are organized on the left side, with nonstructural predicted ORFs on the genome’s right side. These two regions are separated by lysis and immunity cassettes. Some of the structural predicted ORFs include ORF 6 (major capsid protein), ORF 13 (major tail protein), ORF 16 (tape measure protein), and ORFs 17 to 21 (minor tail proteins). The tail assembly chaperones (ORFs 14 and 15) have a −2 frameshift. Like other cluster N phages, Fulbright contains a predicted tyrosine integrase (ORF 35) and immunity repressor (ORF 36) transcribed in the reverse direction. A cluster includes phages with sequence similarity over 50% of their genomes (12). The lysis system is encoded by predicted ORF 27 (lysin A) and ORF 28 (holin) and lacks the lysin B gene. The genome lacks any tRNA genes, which is in line with other phages in this cluster. The phage genome shares 99% nucleotide identity with phage Phloss in the same cluster as that determined using BLASTn.

### Data availability.

The complete genome sequence of phage Fulbright is available in GenBank under the accession number MK977708 with the NCBI SRA accession number SRX10061435.
